# Annotations

**Published:** 1908-11-21

**Authors:** 


### Annotations.

### The Sleep of School Children.

Difficult and obscure as are many of the problems connected with that suspension of consciousness, complete or nearly complete, which we call sleep, there are certain points of personal experience on which most people are agreed. One is that the quantity of daily sleep necessary lessens with advancing age; another is that the brain requires longer rest to recover full vigour than does the mere physical mechanism of the body. Both these points have a strong bearing on the hygiene of the school child, some very important facts concerning whose sleep time were revealed to the Child Study Society last week by Miss Alice Ravenhill. She finds, by returns as to the hours of sleep obtained by over 6,000 children in public elementary schools, that on an average they miss some three hours a day of the sleep suitable and necessary for their ages. Such a state of things is bad enough, but the lecturer further pointed out that even the quality of that which is obtained is very often defective. Overcrowding with its usual accessory, bad ventilation, is one of the factors which is in special need of remedy; another is noise, though it is probable that most town-bred children are too accustomed to this to allow it to affect their sleep. Defective home discipline is also blamed for part of the evil, probably quite correctly. Premature employment, both before and after school hours, is another all too common form of parental selfishness, which is having an important effect in the deterioration of the race. Only a few weeks ago some shocking cases of this were exposed in one of the western suburbs of London; children were compelled to start milk distributing at 5.30 a.m., and even to go on duty again after the completion of their day's school work. Attention was in that case aroused by the teacher's observation that these particular children invariably fell asleep during their lessons. The matter is one upon which public opinion needs education, and in this process the medical profession must assist.

### A Medical Impostor.

In the annals of fraud and false pretences there can be records of but few acts more impudent than that for which W. H. Tucker, *alias* J. Dale and several other names, was fined £15 on November 5. It may be remembered that this individual was condemned in 1906 to three years' penal servitude for numerous offences against the Births and Deaths Registration Acts and for fraud in connection with the sale of a practice in Warwickshire. At that time he was masquerading in the style and title of a gentlemen who practises in the somewhat remote islands of Bermuda and is, therefore, not available to protect himself and the public against such personation. He was released from prison in June last, on license for the unexpired period of his sentence; and he actually renewed his career of unqualified practice under the very same name as before. In September he had the effrontery to approach a medical agent, Mr. Field Hall, with a view to selling the "practice," acquired (since June) in Hackney; and he signed one of the usual forms, on which the details of the practice were set forth, in the name of William Tucker, M.B. Now this man has, speaking broadly, lived for twenty years and more by various frauds in connection with the sale of practices and by personating and forging the signatures of various medical men; for which offences he has been on three occasions convicted in criminal courts. In these circumstances we do not think that a fine is a suitable punishment for his latest offence, and we regret extremely that the stipendiary was unable to commit the prisoner for trial. No doubt the sentence was influenced in some degree by the knowledge that the license will probably be revoked, but if society is to be protected against this dangerous impostor something more than fines and revoked licenses seems to be necessary. The other striking point is that the Medical Defence Union were the prosecutors in the case. In 1906 the Treasury took over from the Union the duty of prosecuting the man, and we do not see why the police should not have done so in the present instance. It is not fair that the Defence Union (that is, ultimately, the medical profession) should have to pay the expenses of convicting a man for breaking the laws of the land.

### The Dangers of Soda Water.

Last year attention was drawn in these columns to the consternation created among the Parisian public by a report on the bacteriological examination of various table-waters, which showed that a large number of them were extremely impure, and contained a relatively much larger proportion of micro-organisms than ordinary tap-water. There is a deep-rooted abhorrence among the French people to the use of tap-water for drinking purposes, and table-waters are extremely popular, owing to the belief that by their use all fear of contamination was avoided. The report was therefore a most unwelcome surprise. London has recently suffered a similar rude awakening. Dr. William Collingridge, Medical Officer of Health for London, has recently publishel a report on the bacteriological examination of soda waters, which shows a similar state of things to that discovered in Paris. Dr. E. E. Klein, who carried out the bacteriological investigations finds that of 36 samples examined, only 27 per cent. were pure, and 52 per cent. were impure, while the rest he designates as "fairly pure." Inspection of the factories showed that contamination was due to imperfections in the bottle-washing and also to improper storage of water in wooden tanks. It is satisfactory to learn at the same time that a large proportion of the factories have undertaken to carry out the necessary alterations in accordance with the recommendations of the Medical Officer, and a register is to be kept of those manufacturers who have complied with their recommendations. It is further proposed that those who fail to comply shall be dealt with according to the provisions of the Sale of Food and Drugs Act.

